# Real‐world safety and effectiveness of adalimumab in patients with pyoderma gangrenosum: Interim analysis of a post‐marketing observational study in Japan

**DOI:** 10.1111/1346-8138.17547

**Published:** 2024-11-13

**Authors:** Toshiyuki Yamamoto, Keiichi Yamanaka, Kenshi Yamasaki, Hisaaki Isaji, Naoko Matsubara, Hiroyuki Hozawa, Tamihiro Kawakami

**Affiliations:** ^1^ Department of Dermatology Fukushima Medical University Fukushima Japan; ^2^ Department of Dermatology Mie University, Graduate School of Medicine Tsu Mie Japan; ^3^ Department of Dermatology Tohoku University Sendai Japan; ^4^ Abbvie GK Tokyo Japan; ^5^ Department of Dermatology Tohoku Medical and Pharmaceutical University Sendai Japan; ^6^ Present address: ALOOP Clinic and Lab Tokyo Japan; ^7^ Present address: Gilead Sciences KK Tokyo Japan

**Keywords:** adalimumab, observational study, post‐marketing product surveillance, pyoderma gangrenosum, safety

## Abstract

Pyoderma gangrenosum (PG) is a rapidly progressive disease characterized by deep ulcers, predominantly in the lower extremities. Adalimumab, a monoclonal antibody against tumor necrosis factor alpha, is the first drug approved for PG treatment in Japan, ahead of other countries. We conducted a multicenter, open‐label, post‐marketing observational study to evaluate the safety and effectiveness of adalimumab in Japanese patients with PG. Of 67 patients enrolled, 37 in the safety analysis set and 32 in the effectiveness analysis set were included in this interim analysis. (Nineteen patients whose case report forms were not collected and 11 whose data were not fixed by the data cut‐off date were excluded from the study). In the safety analysis set, the mean age was 62.9 years and 86.5% of patients had comorbidities, including ulcerative colitis (21.6%), diabetes mellitus (18.9%), and hypertension (10.8%); subtypes of PG included ulcerative (*n* = 33), vegetative (*n* = 2), and pustular (*n* = 2). Mean exposure duration to adalimumab was 185.5 days. Systemic steroids were used before (70.3%) and during (56.8%) adalimumab treatment. The incidence proportion of overall adverse drug reactions was 18.9%. The incidence proportions of all infections and serious infections reported as adverse drug reactions were 13.5% and 10.8%, respectively. The proportion of patients with a Physician Global Assessment score (total lesions) of 0/1 at weeks 12, 26, and 52 was 42.9%, 36.8%, and 50.0%, respectively. This interim analysis revealed the characteristics of Japanese patients with PG treated with adalimumab in the actual clinical setting and the real‐world safety and effectiveness of adalimumab. At the time of the interim analysis, adalimumab treatment was generally well tolerated, and no new safety concerns were detected. Further follow‐up of this study will provide a more detailed understanding of the long‐term safety and effectiveness of adalimumab in patients with PG refractory to conventional treatments.

## 1 INTRODUCTION

Pyoderma gangrenosum (PG) is a rapidly progressive disease characterized by painful single or multiple ulcers, predominantly in the lower extremities.^1^, ^2^, ^3^ PG is clinically classified into several subtypes, with the classic or ulcerative type the most common subtype, present in approximately 85% of cases.^4^ In ulcerative PG, ulcerative lesions with a peripheral inflammatory halo may arise de novo or as a pathergic response to trauma.^1^ Common comorbidities of PG include rheumatoid arthritis, ulcerative colitis, Crohn's disease, acute myeloid leukemia, and Takayasu arteritis.^2^, ^5^ Owing to its non‐specific histological findings, the diagnosis of PG is challenging and often delayed.^6^ In Japan, the estimated incidence of PG is approximately 3 per million per year, and many patients with PG are in their 50s to 70s.^7^, ^8^

The pathophysiology of PG remains poorly understood but involves the predominance of neutrophils in the absence of infection.^9^, ^10^ PG lesions overexpress inflammatory mediators and cytokines, including tumor necrosis factor alpha (TNF‐α).^11^ Adalimumab is a recombinant human immunoglobulin G1 monoclonal antibody that specifically binds to TNF‐α and neutralizes the biological function of TNF by blocking its interaction with the p55 and p75 cell surface TNF receptors.^12^ Previous studies have shown the effectiveness of TNF‐α inhibitors, such as adalimumab and infliximab, for treating PG ulcers.^13^, ^14^, ^15^, ^16^

In November 2020, adalimumab was approved for the treatment of PG in Japan, ahead of other countries, based on the results of an open‐label, single‐arm, multicenter phase 3 study (NCT03311464),^17^, ^18^ which showed that the safety and effectiveness profiles of adalimumab for PG were consistent with those in previous clinical studies for other indications.^19^, ^20^, ^21^, ^22^ At week 26 in the phase 3 study, 54.5% (12/22) of Japanese patients with ulcerative PG had achieved PG area reduction 100 (defined as complete closure of the target PG ulcer), and 54.5% (12/22) had achieved a Physician Global Assessment (PGA) score of 0/1.^17^, ^18^ During the 26‐week treatment period in the phase 3 study, 82% (18/22) patients experienced adverse events (AEs), including infection (50%,  11/22) and a serious AE (18%,  4/22).^17^

To evaluate the real‐world safety and effectiveness of adalimumab in Japanese patients with PG, we conducted a multicenter, open‐label, post‐marketing observational study. We report the interim analysis of the study with a data cut‐off date of December 31, 2022.

## 2 METHODS

### 2.1 Study design, population, and treatment

This multicenter, open‐label, post‐marketing observational study was registered in ClinicalTrials.gov (NCT04750213) and conducted in accordance with the Good Post‐marketing Study Practice (GPSP) (Ministerial Ordinance no. 171 of the Ministry of Health, Labour and Welfare, dated December 20, 2004). Approval from the institutional review board and ethics committee, which is not required in GPSP, was obtained if requested by the participating medical institutions.

Patients were enrolled in the study between November 27, 2020 and January 31, 2023. Patients diagnosed with PG and prescribed adalimumab for the first time in routine practice by the treating physician were registered in a central registration system, after providing consent. Patients who had been previously treated with adalimumab for PG were excluded from the study; patients who had been previously treated with adalimumab for other indications were not excluded from the study. We set the target sample size at 60 patients, based on the following assumptions and considerations for possible loss to follow‐up: (i) the incidence proportion of adalimumab‐related infection in patients with PG in the previous study was 18.2%,^18^ and (ii) at least 58 participants were required to estimate the incidence proportion within the range of 8.2% to 28.2% (mean [SD] 18.2% [± 10.0%]) with an alpha level of 0.05.

Eligible patients were treated via subcutaneous injection of adalimumab, according to the package insert.^23^ The first dose was 160 mg; the second dose was 80 mg, 2 weeks after the first dose; and the third dose was 40 mg at 4 weeks after the first dose, and 40 mg once a week thereafter. Patients were observed for 52 weeks after the first dose, and those who discontinued adalimumab before week 52 were followed up for 70 days after the last dose.

### 2.2 Study items and data collection

After the first injection of adalimumab, treating physicians filled out the registration form within 14 days, with the injection date as day 0. Treating physicians then submitted case report forms via an internet‐based electronic data capture system at weeks 26 and 52 or at adalimumab discontinuation. The sponsor reviewed the data submitted in the case report forms and conducted inquiries, as necessary.

The following data were collected from medical records at the participating institutions: baseline patient characteristics including age, sex, weight, pregnancy and lactation status, comorbidities, medical history, prior treatment with TNF inhibitors for conditions other than PG, disease duration of PG, number of PG lesions, PG subtype (ulcerative [including peristomal], bullous, pustular, or vegetative), affected site, target lesion (defined as the largest lesion of each subtype) and its size, and previous treatment for PG. The administration status of adalimumab, concomitant treatment for PG, and AEs were recorded throughout the study period. AEs were considered adverse drug reactions (ADRs) if a causal relationship with adalimumab treatment could not be ruled out. Disease severity was evaluated at weeks 12, 26, and 52 (or at discontinuation) on a 7‐point PGA scale, with 0 for completely clear and 6 for worse.^24^ Erythema and border elevation severity was evaluated at weeks 0, 12, 26, and 52 (or at discontinuation) on a 5‐point Investigator Inflammation Assessment (IIA) scale, with 0 for none and 4 for very severe.^25^ The pain level was evaluated at weeks 0, 12, 26, and 52 (or at discontinuation) using a 4‐point Verbal Rating Scale (VRS), with 0 for no pain and 3 for severe pain.^26^

### 2.3 Endpoints

The primary endpoint was the incidence proportion of all infections reported as ADRs during the 52‐week observation period. The secondary endpoints were the incidence proportion of serious infections reported as ADRs and the incidence proportion of ADRs other than infections during the observation period. The effectiveness endpoints were the PGA scores (total lesions) at weeks 12, 26, and 52 (or at discontinuation), IIA scores (for the target lesion) at weeks 0, 12, 26, and 52 (or at discontinuation), and pain VRS scores at weeks 0, 26, and 52 (or at discontinuation).

### 2.4 Statistical analysis

Descriptive statistics were used to describe the patients and PG disease characteristics. AEs and ADRs were coded to primary system organ classes and preferred terms in a Japanese edition of the Medical Dictionary for Regulatory Activities (version 25.1). The proportions of patients with a PGA score of 0/1 or 0 (total lesions and the target lesion) at weeks 12, 26, and 52 and at treatment discontinuation are presented with 95% Clopper–Pearson confidence intervals (CIs). The proportions of patients with an IIA score of 0 (erythema and border elevation at the target lesion) at weeks 0, 12, 26, and 52 and at treatment discontinuation are presented with 95% CIs. The proportions of patients with a pain VRS score of 0 at weeks 0, 26, and 52 and at treatment discontinuation are presented with 95% CIs. Pain VRS scores at weeks 0, 26, and 52 and at treatment discontinuation are presented as mean (standard deviation [SD]). Missing data were not imputed. Statistical analysis was performed using SAS software version 9.4 (SAS Institute Inc., Cary, NC, USA).

## 3 RESULTS

### 3.1 Baseline patient characteristics

**FIGURE 1:**
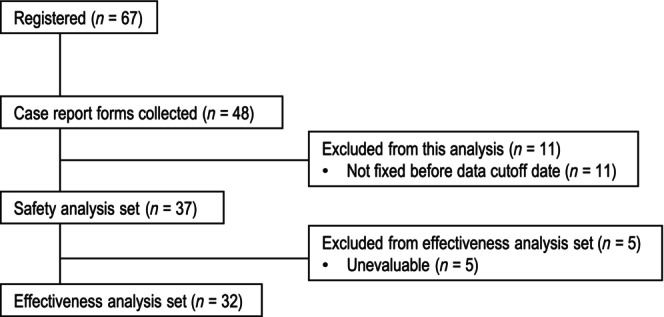
Patient disposition.

Of 67 patients registered at 54 sites in Japan, case report forms were collected from 48 patients for this interim analysis (Figure 1). After excluding 11 patients whose data were not fixed by the data cut‐off date (December 31, 2022), 37 patients were included in the safety analysis set. After excluding five unevaluable patients, 32 patients were included in the effectiveness analysis set.

In the safety analysis set, the mean (SD) age was 62.9 (16.4) years, with more male (*n* = 21) than female (*n* = 16) patients. Mean (SD) patient weight was 60.96 (12.92) kg, 86.5% (32/37) of patients had comorbidities, and 27.0% (10/37) had a medical history at baseline (Tables 1, S1, and S2). The most common comorbidities included ulcerative colitis (21.6% [8/37]), diabetes mellitus (18.9% [7/37]), and hypertension (10.8% [4/37]). TNF inhibitors, specifically infliximab or adalimumab, had been used previously in 10.8% (4/37) of patients for conditions other than PG: Crohn's disease (infliximab, *n* = 2) and ulcerative colitis (infliximab, *n* = 1; adalimumab, *n* = 1).

**TABLE 1:** Baseline patient characteristics.

	Safety analysis set *N* = 37	Effectiveness analysis set *N* = 32
Age, years		
Mean (SD)	62.9 (16.4)	62.5 (16.7)
Median (range)	65.0 (17–83)	64.5 (17–83)
Sex		
Male	21 (56.8)	17 (53.1)
Female	16 (43.2)	15 (46.9)
Weight, kg		
*n*	30	26
Mean (SD)	60.96 (12.92)	61.68 (13.72)
Median (range)	57.85 (45.0–95.0)	58.50 (45.0–95.0)
Pregnancy^a^		
No	36 (97.3)	32 (100.0)
Unknown/not recorded	1 (2.7)	0 (0.0)
Lactation^b^		
No	16 (100.0)	15 (100.0)
Comorbidities		
Yes	32 (86.5)	29 (90.6)
Common comorbidities (≥3 in safety analysis set)		
Ulcerative colitis	8 (21.6)	7 (21.9)
Diabetes mellitus	7 (18.9)	7 (21.9)
Hypertension	4 (10.8)	4 (12.5)
Crohn's disease	3 (8.1)	3 (9.4)
Rheumatoid arthritis	3 (8.1)	3 (9.4)
Type 2 diabetes mellitus	3 (8.1)	2 (6.3)
Medical history		
Yes	10 (27.0)	9 (28.1)
Prior treatment with TNF inhibitors for conditions other than PG		
Yes	4 (10.8)	3 (9.4)
Ulcerative colitis	2 (5.4)	1 (3.1)
Crohn's disease	2 (5.4)	2 (6.3)

*Note*: Values are presented as *n* (%) unless otherwise indicated.

Abbreviations: PG, pyoderma gangrenosum; SD, standard deviation; TNF, tumor necrosis factor.

^a^ Partner's pregnancy status for male patients.

^b^ For female patients.

### 3.2 Baseline PG disease characteristics

In the safety analysis set, 81.1% (30/37) of patients had had PG for 1 to <3 years, with a mean (SD) disease duration of 2.7 (5.0) years, and 89.2% (33/37) of patients had 1 to 5 lesions (mean [SD] of 5.4 [16.2]) (Table 2). The ulcerative type (including the peristomal type) was the most common PG subtype, found in 89.2% (33/37) of patients. The most commonly affected sites were the lower limbs (83.8% [31/37]) and the trunk (10.8% [4/37]), and the most common sites of target lesions were the lower limbs (78.4% [29/37]) and artificial anus (8.1% [3/37]). The mean (SD) size of the target lesion was 80.1 (63.5) mm.

**TABLE 2:** Baseline pyoderma gangrenosum (PG) characteristics.

	Safety analysis set *N* = 37	Effectiveness analysis set *N* = 32
Disease duration, years		
1 to <3	30 (81.1)	26 (81.3)
3 to <5	1 (2.7)	0 (0.0)
≥5	4 (10.8)	4 (12.5)
Unknown/not recorded	2 (5.4)	2 (6.3)
Mean (SD)	2.7 (5.0)	2.9 (5.3)
Median (range)	1.0 (1.0–25.0)	1.0 (1.0–25.0)
Number of PG lesions		
1 to 5	33 (89.2)	28 (87.5)
6 to 10	2 (5.4)	2 (6.3)
11 to 15	0 (0.0)	0 (0.0)
16 to 20	1 (2.7)	1 (3.1)
≥21	1 (2.7)	1 (3.1)
Mean (SD)	5.4 (16.2)	6.0 (17.3)
Median (range)	2.0 (1–99)	2.0 (1–99)
PG subtype		
Ulcerative including peristomal	33 (89.2)	28 (87.5)
Ulcerative excluding peristomal	30 (81.1)	27 (84.4)
Peristomal	3 (8.1)	1 (3.1)
Pustular	2 (5.4)	2 (6.3)
Vegetative	2 (5.4)	2 (6.3)
PG‐affected site^a^		
Lower limbs	31 (83.8)	29 (90.6)
Trunk	4 (10.8)	3 (9.4)
Artificial anus	3 (8.1)	1 (3.1)
Upper limbs	3 (8.1)	3 (9.4)
Genital	1 (2.7)	1 (3.1)
Other	1 (2.7)	1 (3.1)
Site of target lesion		
Lower limbs	29 (78.4)	27 (84.4)
Artificial anus	3 (8.1)	1 (3.1)
Trunk	2 (5.4)	1 (3.1)
Upper limbs	1 (2.7)	1 (3.1)
Genital	1 (2.7)	1 (3.1)
Other	1 (2.7)	1 (3.1)
Size of target lesion (major axis), mm		
*n*	36	32
Mean (SD)	80.1 (63.5)	80.1 (62.3)
Median (range)	71.5 (4–300)	76.5 (4–300)
IIA score (0–4)		
*n*	32	30
Moderate to very severe erythema (2–4)	24 (75.0)	22 (73.3)
Moderate to very severe border elevation (2–4)	21 (65.6)	21 (70.0)
Pain VRS score (0–3)		
*n*	–	28
Moderate to severe pain (2, 3)	–	17 (60.7)
Mean (SD)	–	1.82 (0.77)

*Note*: Values are presented as *n* (%) unless otherwise indicated.

Abbreviations: IIA, investigator inflammation assessment; SD, standard deviation; VRS, verbal rating scale.

^a^ Multiple answers can be selected.

In the safety analysis set, 75.0% (24/32) of patients had moderate to very severe erythema with an IIA score of 2 to 4, and 65.6% (21/32) had moderate to very severe border elevation with an IIA score of 2 to 4. In the effectiveness analysis set, 60.7% (17/28) of patients had moderate to severe pain, with a pain VRS score of 2 to 3 at baseline, and a mean (SD) score of 1.82 (0.77).

### 3.3 Administration status of adalimumab

The mean (SD) and median (range) duration of adalimumab treatment was 185.5 (119.1) and 183.0 (1–365) days, respectively, and 83.8% (31/37) of patients were treated with the dosage per the prescribing information (Table 3). By the data cut‐off date, 45.9% (17/37) of patients in the safety analysis set and 40.6% (13/32) in the effectiveness analysis set had discontinued adalimumab treatment. The reasons for discontinuation included AEs (47.1% [8/17] in the safety analysis set, 46.2% [6/13] in the effectiveness analysis set), loss to follow‐up (including hospital transfers) (23.5% [4/17] in the safety analysis set, 23.1% [3/13] in the effectiveness analysis set), symptom improvement (17.6% [3/17] in the safety analysis set, 23.1% [3/13] in the effectiveness analysis set), and low effectiveness (17.6% [3/17] in the safety analysis set, 23.1% [3/13] in the effectiveness analysis set).

**TABLE 3:** Administration status of adalimumab.

Treatment	Safety analysis set *N* = 37	Effectiveness analysis set *N* = 32
Duration of adalimumab treatment^a^		
<4 weeks	4 (10.8)	3 (9.4)
4 to <12 weeks	4 (10.8)	1 (3.1)
12 to <26 weeks	5 (13.5)	5 (15.6)
26 to <52 weeks	16 (43.2)	16 (50.0)
52 weeks	8 (21.6)	7 (21.9)
Mean (SD), days	185.5 (119.1)	197.8 (112.3)
Median (range), days	183.0 (1–365)	183.0 (1–365)
Dosage		
Per prescribing information	31 (83.8)	27 (84.4)
Other	6 (16.2)	5 (15.6)
Discontinuation of adalimumab treatment		
Yes	17 (45.9)	13 (40.6)
Reason for discontinuation^b^		
Adverse events (including death)	8 (47.1)	6 (46.2)
Loss to follow‐up (including hospital transfers)	4 (23.5)	3 (23.1)
Symptom improvement	3 (17.6)	3 (23.1)
Low effectiveness	3 (17.6)	3 (23.1)
Other	1 (5.9)	0 (0.0)

*Note*: Values are presented as *n* (%) unless otherwise indicated.

Abbreviation: SD, standard deviation.

^a^ Period included temporarily suspended duration.

^b^ Relative to the number of discontinued patients; multiple reasons can be chosen.

### 3.4 Treatment for PG before and during the study

Before initiating adalimumab treatment, all (37/37) patients in the safety analysis set had received pharmacological treatment for PG, and 13.5% (5/37) of patients had received non‐pharmacological treatment for PG, including surgery (8.1% [3/37]) (Table 4). Common systemic therapy included steroids (70.3% [26/37]), analgesics such as non‐steroidal anti‐inflammatory drugs (35.1% [13/37]), antibiotics/antibacterial drugs (18.9% [7/37]), and cyclosporine (13.5% [5/37]). Common local therapies included steroids (48.6% [18/37]), debridement agents (24.3% [9/37]), tissue regeneration accelerators (16.2% [6/37]), and antibiotics/antibacterial drugs (16.2% [6/37]). Most previous medications for PG had been used until immediately before adalimumab initiation (Table S3).

**TABLE 4:** Treatment for pyoderma gangrenosum (PG) before and during the study.

Treatment	Safety analysis set *N* = 37
Previous treatment	
Pharmacological treatment for PG	37 (100.0)
Systemic therapy^a^	
Steroids	26 (70.3)
Analgesics (e.g., NSAIDs)	13 (35.1)
Antibiotics/antibacterial drugs	7 (18.9)
Cyclosporine	5 (13.5)
Local therapy^a^	
Steroids	18 (48.6)
Debridement agents	9 (24.3)
Tissue regeneration accelerators	6 (16.2)
Antibiotics/antibacterial drugs	6 (16.2)
Non‐pharmacological treatment for PG	5 (13.5)
Surgery	3 (8.1)
Other	2 (5.4)
Concomitant treatment	
Pharmacological treatment for PG	33 (89.2)
Systemic therapy^a^	
Steroids	21 (56.8)
Analgesics (e.g., NSAIDs)	10 (27.0)
Local therapy^a^	
Steroids	9 (24.3)
Antibiotics/antibacterial drugs	6 (16.2)
Non‐pharmacological treatment for PG	4 (10.8)
Surgery	1 (2.7)
Negative pressure wound therapy	1 (2.7)
Other	4 (10.8)

*Note*: Values are presented as *n* (%).

Abbreviation: NSAIDs, nonsteroidal anti‐inflammatory drugs.

^a^ Therapy used in ≥5 patients.

During adalimumab treatment, 89.2% (33/37) of patients in the safety analysis set received concomitant pharmacological treatment for PG, and 10.8% (4/37) received non‐pharmacological treatment for PG, including surgery (2.7% [1/37]) and negative pressure wound therapy (2.7% [1/37]) (Table 4). Common systemic therapy included steroids (56.8% [21/37]) and analgesics (27.0% [10/37]). The most common local therapies included steroids (24.3% [9/37]), antibiotics/antibacterial drugs (16.2% [6/37]). Most concomitant medications for PG had been initiated before adalimumab initiation (Table S4).

### 3.5 Safety of adalimumab

The incidence proportion of infections reported as ADRs was 13.5% (seven events in 5/37 of patients): pneumonia and tuberculosis (*n* = 1), sepsis (*n* = 1), urinary tract infection (*n* = 1), pneumonia and cytomegalovirus enterocolitis (*n* = 1), and COVID‐19 (*n* = 1) (Table 5). The incidence proportion of infections reported as serious ADRs was 10.8% (five events in 4/37 of patients): tuberculosis (*n* = 1), sepsis (*n* = 1), urinary tract infection (*n* = 1), and pneumonia and cytomegalovirus enterocolitis (*n* = 1). A serious ADR other than infection, cerebral infarction occurred in one patient with pneumonia and cytomegalovirus enterocolitis ADRs.

**TABLE 5:** Summary of adverse events and adverse drug reactions (safety analysis set, *N* = 37).

	AEs	Serious AEs	ADRs	Serious ADRs
Incidence proportion	15 (40.5)	11 (29.7)	7 (18.9)	4 (10.8)
Number of events	21	14	11	6
Infections and infestations	8 (21.6)	6 (16.2)	5 (13.5)	4 (10.8)
Pneumonia	3 (8.1)	2 (5.4)	2 (5.4)	1 (2.7)
Cellulitis	1 (2.7)	0 (0.0)	0 (0.0)	0 (0.0)
Pneumonia aspiration	1 (2.7)	1 (2.7)	0 (0.0)	0 (0.0)
Sepsis	1 (2.7)	1 (2.7)	1 (2.7)	1 (2.7)
Tuberculosis	1 (2.7)	1 (2.7)	1 (2.7)	1 (2.7)
Urinary tract infection	1 (2.7)	1 (2.7)	1 (2.7)	1 (2.7)
Cytomegalovirus enterocolitis	1 (2.7)	1 (2.7)	1 (2.7)	1 (2.7)
COVID‐19	1 (2.7)	0 (0.0)	1 (2.7)	0 (0.0)
Benign, malignant and unspecified neoplasms (including cysts and polyps)				
Bladder cancer recurrent	1 (2.7)	1 (2.7)	0 (0.0)	0 (0.0)
Nervous system disorders				
Cerebral infarction	1 (2.7)	1 (2.7)	1 (2.7)	1 (2.7)
Cardiac disorders				
Cardiac failure	1 (2.7)	1 (2.7)	0 (0.0)	0 (0.0)
Vascular disorders				
Aortic dissection	1 (2.7)	1 (2.7)	0 (0.0)	0 (0.0)
Gastrointestinal disorders				
Gastrointestinal hemorrhage	1 (2.7)	1 (2.7)	0 (0.0)	0 (0.0)
Hepatobiliary disorders				
Abnormal hepatic function	1 (2.7)	0 (0.0)	1 (2.7)	0 (0.0)
Skin and subcutaneous tissue disorders				
Pyoderma gangrenosum	2 (5.4)	2 (5.4)	0 (0.0)	0 (0.0)
Dermatitis contact	1 (2.7)	0 (0.0)	0 (0.0)	0 (0.0)
Palmoplantar pustulosis	1 (2.7)	0 (0.0)	1 (2.7)	0 (0.0)
Musculoskeletal and connective tissue disorders				
Bursitis	1 (2.7)	0 (0.0)	1 (2.7)	0 (0.0)

*Note*: Values are presented as *n* (%) or *n*. Adverse events (AEs) and adverse drug reactions (ADRs) were coded to primary system organ classes and preferred terms in a Japanese version of the Medical Dictionary for Regulatory Activities (version 25.1).

Abbreviation: COVID‐19, coronavirus disease 2019.

The incidence proportion of overall AEs and ADRs was 40.5% (21 events in 15/37 patients) and 18.9% (11 events in 7/37 patients), respectively (Table 5). The incidence proportion of ADRs leading to adalimumab discontinuation was 13.5% (eight events in 5/37 patients): pneumonia, cytomegalovirus enterocolitis, and cerebral infarction (*n* = 1), pneumonia and tuberculosis (*n* = 1), sepsis (*n* = 1), urinary tract infection (*n* = 1), and abnormal hepatic function (*n* = 1) (Table S5).

Two patients died during this study. One patient was treated with adalimumab for 57 days and developed aortic dissection 70 days after the first dose; the patient's death was considered unrelated to adalimumab treatment. The other patient was treated with adalimumab for 1 day and developed pneumonia, cytomegalovirus enterocolitis, and cerebral infarction 12 to 13 days after the first dose. The treating physician considered that this patient's death was due to an immunocompromised state (use of methotrexate for rheumatoid arthritis and corticosteroids for PG) and was unrelated to adalimumab, but the sponsor could not rule out a causal relationship between the death and adalimumab treatment.

### 3.6 Effectiveness of adalimumab

**FIGURE 2:**
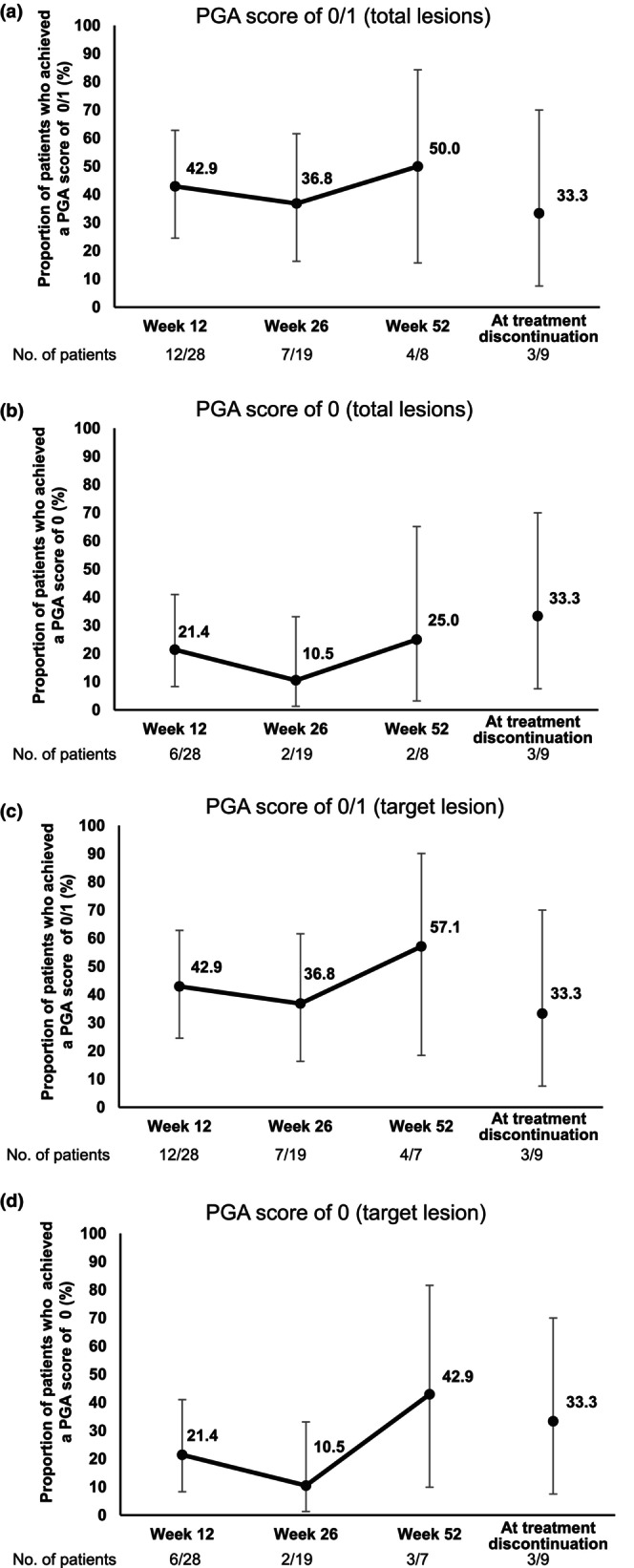
Graphs showing the proportions of patients who achieved a Physician Global Assessment (PGA) score (total lesions) of 0/1 (a) and 0 (b) and patients who achieved a PGA score (target lesion) of 0/1 (c) and 0 (d), with 95% confidence intervals.

The proportion of patients who achieved a PGA score of 0/1 for total lesions was 42.9% (95% CI 24.5–62.8) at week 12, 36.8% (95% CI 16.3–61.6) at week 26, 50.0% (95% CI 15.7–84.3) at week 52, and 33.3% (95% CI 7.5–70.1) at treatment discontinuation (Figure 2a). The proportion of patients who achieved a PGA score of 0 for total lesions was 21.4% (95% CI 8.3–41.0) at week 12, 10.5% (95% CI 1.3–33.1) at week 26, 25.0% (95% CI 3.2–65.1) at week 52, and 33.3% (95% CI 7.5–70.1) at treatment discontinuation (Figure 2b). Achievement of a PGA score of 0/1 for total lesions was observed across subtypes (Table S6). The proportion of patients who achieved a PGA score of 0/1 for the target lesion was 42.9% (95% CI, 24.5–62.8) at week 12, 36.8% (95% CI 16.3–61.6) at week 26, 57.1% (95% CI 18.4–90.1) at week 52, and 33.3% (95% CI 7.5–70.0) at treatment discontinuation (Figure 2c). The proportion of patients who achieved a PGA score of 0 for the target lesion was 21.4% (95% CI 8.3–41.0) at week 12, 10.5% (95% CI 1.3–33.1) at week 26, 42.9% (95% CI 9.9–81.6) at week 52, and 33.3% (95% CI 7.5–70.1) at treatment discontinuation (Figure 2d). When patients with lesions ≥100 mm and those <100 mm were analyzed separately, the proportion of patients who achieved a PGA score of 0/1 was numerically higher in patients with lesions ≥100 mm in the major axis than those with lesions <100 mm at weeks 12 and 26, but not at week 52 (total lesions, Figure S1a; the target lesion, Figure S1b).

**FIGURE 3:**
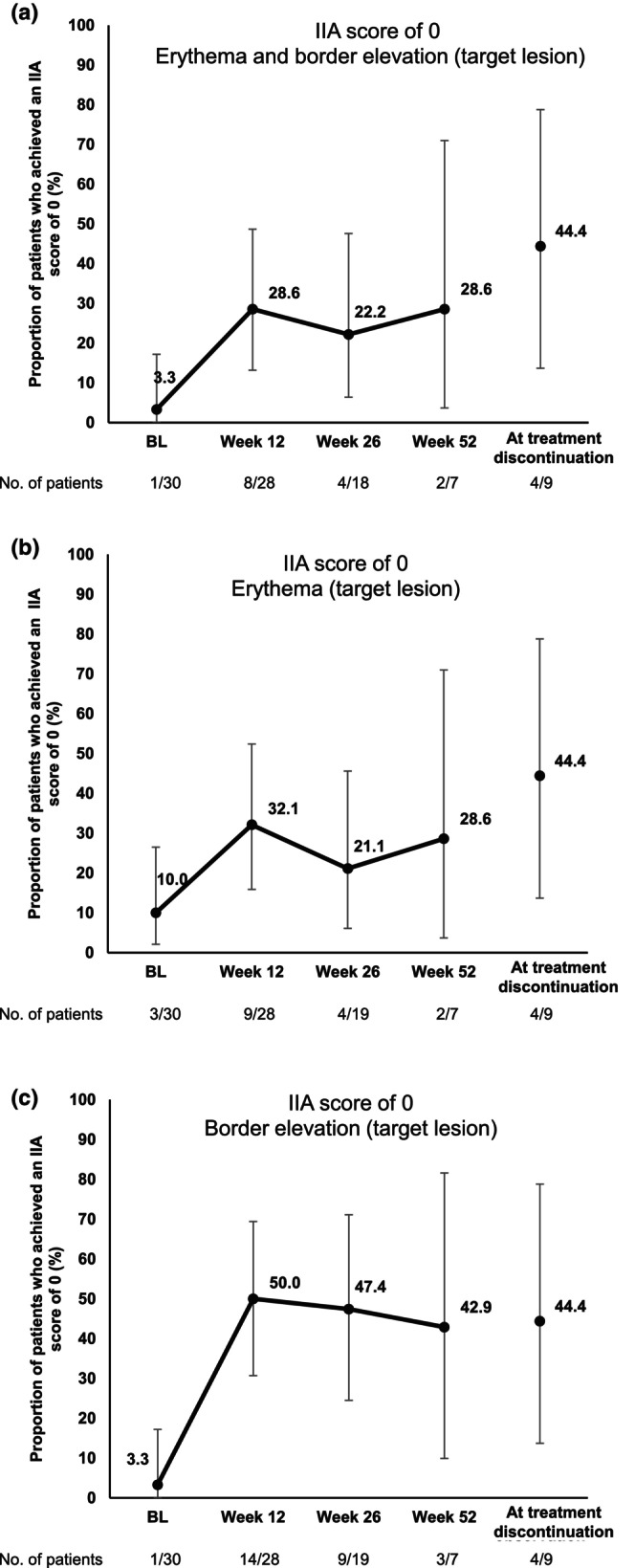
Graphs showing the proportions of patients who achieved an Investigator Inflammation Assessment (IIA) score (target lesion) of 0 for (a) erythema and border elevation, (b) erythema and (c) border elevation (c), with 95% confidence intervals. BL, baseline.

The proportion of patients who achieved an IIA score of 0 for erythema and border elevation (target lesion) was 3.3% (95% CI 0.1–17.2) at baseline, 28.6% (95% CI 13.2–48.7) at week 12, 22.2% (95% CI 6.4–47.6) at week 26, 28.6% (95% CI 3.7–71.0) at week 52, and 44.4% (95% CI 13.7–78.8) at treatment discontinuation (Figure 3a). The proportion of patients who achieved an IIA score of 0 for erythema alone (target lesion) was 10.0% (95% CI 2.1–26.5) at baseline, 32.1% (95% CI 15.9–52.4) at week 12, 21.1% (95% CI 6.1–45.6) at week 26, 28.6% (95% CI 3.7–71.0) at week 52, and 44.4% (95% CI 13.7–78.8) at treatment discontinuation (Figure 3b). The proportion of patients who achieved an IIA score of 0 for border elevation (target lesion) was 3.3% (95% CI 0.1–17.2) at baseline, 50.0% (95% CI 30.6–69.4) at week 12, 47.4% at week 26 (95% CI 24.4–71.1), 42.9% (95% CI 9.9–81.6) at week 52, and 44.4% (95% CI 13.7–78.8) at treatment discontinuation (Figure 3c). When patients with lesions ≥100 mm and lesions of <100 mm were analyzed separately, the proportion of patients who achieved an IIA score of 0 was numerically higher in patients with lesions ≥100 mm in the major axis than those with lesions <100 mm at baseline and week 12, but not at weeks 26 and 52 (erythema and border elevation [target lesion], Figure S2; erythema [target lesion], Figure S2; border elevation [target lesion], Figure S2).

**FIGURE 4:**
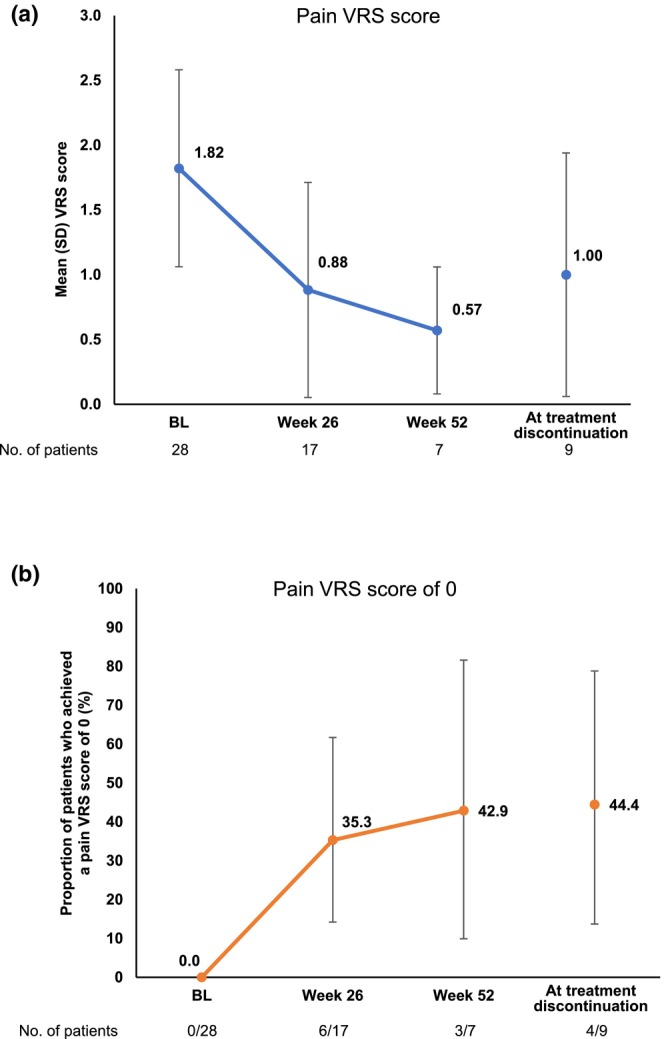
Graphs showing the mean (standard deviation [SD]) pain Verbal Rating Scale (VRS) score (a) and the proportion of patients who achieved a pain VRS score of 0, with 95% confidence intervals (b). BL, baseline.

The mean (SD) pain VRS score was 1.82 (0.77) at baseline, 0.88 (0.86) at week 26, 0.57 (0.53) at week 52, and 1.00 (1.00) at treatment discontinuation (Figure 4a). The proportion of patients who achieved a pain VRS score of 0 was 0.0% at baseline, 35.3% (95% CI 14.2–61.7) at week 26, 42.9% (95% CI 9.9–81.6) at week 52, and 44.4% (95% CI 13.7–78.8) at treatment discontinuation (Figure 4b). When patients with lesions ≥100 mm and <100 mm were analyzed separately, at week 52, the mean pain VRS score was numerically lower (Figure S3) and the proportion of patients who achieved a pain VRS score of 0 was numerically higher (Figure S3) in patients with lesions <100 mm in the major axis than those with lesions ≥100 mm.

## 4 DISCUSSION

This interim analysis of our post‐marketing observational study revealed the patient characteristics and real‐world safety and effectiveness of adalimumab in Japanese patients with PG, including those with PG subtypes other than the ulcerative type. The incidence proportion of all infections reported as ADRs during the 52‐week observation period was 13.5%, which was comparable to that in the phase 3 study,^17^, ^18^ and the proportion of patients with a PGA score (total lesions) of 0/1 at week 26 and week 52 was 36.8% and 50.0%, respectively.

The patient characteristics in the present study were similar to those previously reported in Japan, except for the sex ratio.^7^, ^8^ In Japan, PG is typically observed among people in their 50s to 70s, and it is more prevalent in women, with a sex ratio of 1:1.3 to 1:1.4.^7^, ^8^ In this study, the mean age of included patients was 62.9 years, but there were more male patients than female patients, with a sex ratio of 1.3:1. Consistent with previous reports in Japan^7^, ^8^ and review articles,^1^, ^2^, ^3^ patients in this study had concomitant inflammatory bowel disease, such as ulcerative colitis (21.6%) and Crohn's disease (8.1%), and most patients had ulcerative‐type PG (89.2%). One patient had used adalimumab prior to this study to treat ulcerative colitis and experienced a favorable response to adalimumab in this study. The patient achieved a PGA score (total lesions) of 1 at weeks 12 and 26, a VRS score of 1 at baseline that improved to 0 by week 26, and no AEs during the 26‐week observation period. Other common comorbidities observed among patients in this study included diabetes mellitus (18.9%), hypertension (10.8%), type 2 diabetes mellitus (8.1%), and rheumatoid arthritis (8.1%). Patients in this real‐world study commonly used systemic and local steroids as previous and concomitant treatments, which is in line with the PG treatment guidelines developed by the Japanese Dermatological Association.^27^

In this study, four patients (10.8%) experienced six serious ADRs: tuberculosis (*n* = 1), sepsis (*n* = 1), urinary tract infection (*n* = 1), and pneumonia, cytomegalovirus enterocolitis and cerebral infarction (*n* = 1). Three of these patients had type 2 or steroid‐induced diabetes, one had end‐stage renal disease, and one was treated with methotrexate for rheumatoid arthritis and concomitantly treated with corticosteroids for PG. The treating physicians assumed that a baseline immunocompromised state owing to comorbidities and treatments might have predisposed these patients to serious ADRs that were not related to adalimumab treatment.^28^, ^29^, ^30^, ^31^ In this study, the causal relationship between adalimumab treatment and death was not ruled out in one patient who died of pneumonia, cytomegalovirus enterocolitis, and cerebral infarction. In the phase 3 study of adalimumab among 22 patients with PG, four patients (18%) experienced four serious AEs (anemia, bacterial arthritis, cataract, and pain owing to PG), but no patients died or developed opportunistic infection after the initiation of adalimumab.^17^

In the present study, the proportion of patients who achieved a PGA score of 0/1 (total lesions) was 36.8% at week 26 and 50.0% at week 52. Although a direct comparison cannot be made between studies, these values are slightly lower than those in the previous phase 3 study of adalimumab, in which the proportion of patients who achieved a PGA score of 0/1 (total lesions) was 44.4% at week 26 and 66.7% at week 52.^17^, ^18^ The mean pain score on the 4‐point VRS scale, which was 1.82 at baseline, decreased to 0.88 at week 26 and 0.57 at week 52, suggesting the effectiveness of adalimumab in reducing pain associated with PG. This was similarly shown in the phase 3 study as a decrease in the mean pain score using a numeric rating scale.^18^

The present study included a more diverse patient population than the phase 3 study:^17^, ^18^ patients in our study (i) had PG subtypes other than the ulcerative type (pustular type [*n* = 2] and vegetative type [*n* = 2]), (ii) did not require that the dose of systemic steroids be reduced to a prednisolone equivalent of <10 mg/day (the dose specified in the phase 3 study) before the initiation of adalimumab, and (iii) were allowed to have a target PG lesion of ≥100 mm (only <100 mm in the major axis was allowed in the phase 3 study). Owing to a small sample size, we did not have sufficient data to conclusively determine which factors contributed to the numerically lower proportion of patients who achieved a PGA score of 0/1 (total lesions) at weeks 26 and 52 in the present study compared with the phase 3 study. Further studies are needed to assess the effectiveness of adalimumab in different PG subtypes and the optimal tapering of concomitant systemic steroids.

This study has some limitations. First, the present study included a small number of patients with PG, owing to the rare nature of this disease, which did not allow us to perform a robust statistical analysis. Second, the data in this interim analysis might be skewed owing to the data collection method, that is, data were collected earlier from discontinued patients than those who continued the study. Nonetheless, we believe that rapid public communication of the safety and effectiveness of adalimumab and the characteristics of the patients treated with adalimumab in real‐world settings, even as an interim report, is beneficial to the dermatology community.

In conclusion, this interim analysis of a post‐marketing observational study clarified the real‐world safety and effectiveness of adalimumab and the characteristics of Japanese patients with PG. Adalimumab treatment was generally well tolerated, and no new safety concerns were detected at the time of the interim analysis. Further follow‐up of this study will provide a more detailed understanding of the long‐term safety and effectiveness of adalimumab in patients with PG refractory to conventional treatments.

## FUNDING INFORMATION

This study was sponsored by AbbVie GK, manufacturer and licensee of adalimumab. AbbVie was involved in the trial design, research, analysis, data collection, data interpretation, as well as review and approval of the manuscript for publication. All authors had access to the relevant data and participated in the drafting, review, and approval of this publication. There were no payments or compensations made to the authors for their contributions to this manuscript. Medical writing support was provided by Yuka Kinoshita (ASCA Corporation) in the development of this publication and funded by AbbVie GK.

## CONFLICT OF INTEREST STATEMENT

Keiichi Yamanaka received research grants from Eli Lilly Japan, Leo Pharma, Maruho, Sasaki Chemical, Sun Pharmaceutical Industries, Taiho Pharmaceutical, Torii Pharmaceutical, KAKENHI, Nihon Kayaku, Kaken Pharmaceutical, Nihon Seiyaku, Boehringer Ingelheim, and Otsuka Pharmaceutical; lecture fees from AbbVie, Otsuka Pharmaceutical, Astellas Seiyaku, Boehringer Ingelheim, Pfizer, Celgene, UCB Japan, Daiichi‐Sankyo, Bristol Myers, Eisai, Kaken Pharmaceutical, Eli Lilly Japan, Nobel Pharma, Janssen, Kyowa Kirin, Leo Pharma, Maruho, Mitsubishi Tanabe, Nippon Kayaku, Nippon Zouki, Novartis, Sato Seiyaku, Sanofi, Sun Pharmaceutical Industries, Taiho Pharmaceutical, and Torii Pharmaceutical. Kenshi Yamasaki received research grants from Abbvie during the conduct of the study; received research grants from Kao Corporation and Estee Lauder; consulting fees from Pola Chemical Corporation; and honoraria for lectures from Maruho, Sato Pharmaceutical, Eli Lilly Japan, UCB Japan, Abbvie, Taiho Pharmaceutical, Sun Pharma, Novartis, Eisai, Boehringer Ingelheim Japan, Bristol Myers, Grapha Laboratories, and Sanofi. Hisaaki Isaji is a former employee of Abbvie GK. Naoko Matsubara and Hiroyuki Hozawa are employees of Abbvie GK. Tamihiro Kawakami has nothing to declare.

Toshiyuki Yamamoto, Keiichi Yamanaka, and Kenshi Yamasaki are members of the Editorial Board of The Journal of Dermatology. To minimize bias, they were excluded from all editorial decision‐making related to the acceptance of this article for publication.

## Supporting information


Figure S1.

Figure S2.

Figure S3.

Table S1.
Table S2.
Table S3.
Table S4.
Table S5.
Table S6.

## Data Availability

The datasets generated and/or analyzed during the current study are available from the corresponding author on reasonable request.
