# Supplementary figures and images for: Real‐world safety and effectiveness of adalimumab in patients with pyoderma gangrenosum: Interim analysis of a post‐marketing observational study in Japan
Source: J Dermatol. 2024 Nov 13;52(2):270–80. doi: 10.1111/1346-8138.17547 (PMC11807368; doi:10.1111/1346-8138.17547)

Fig S1.

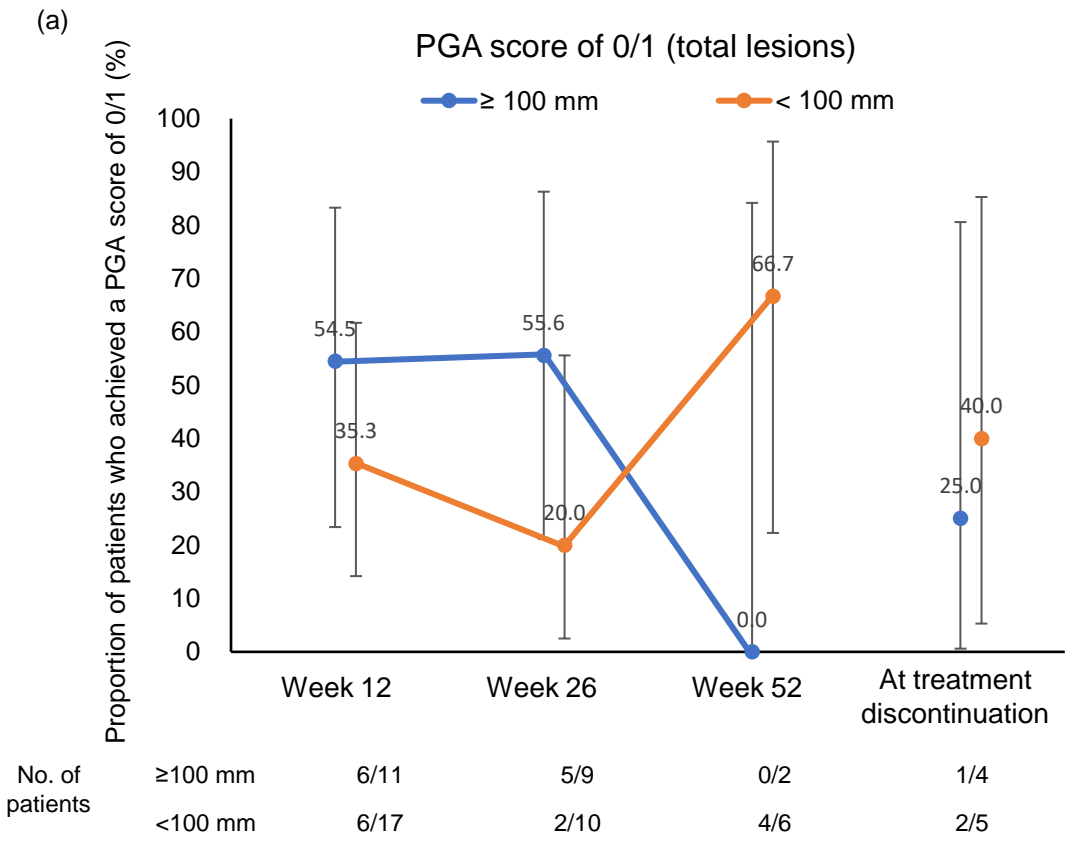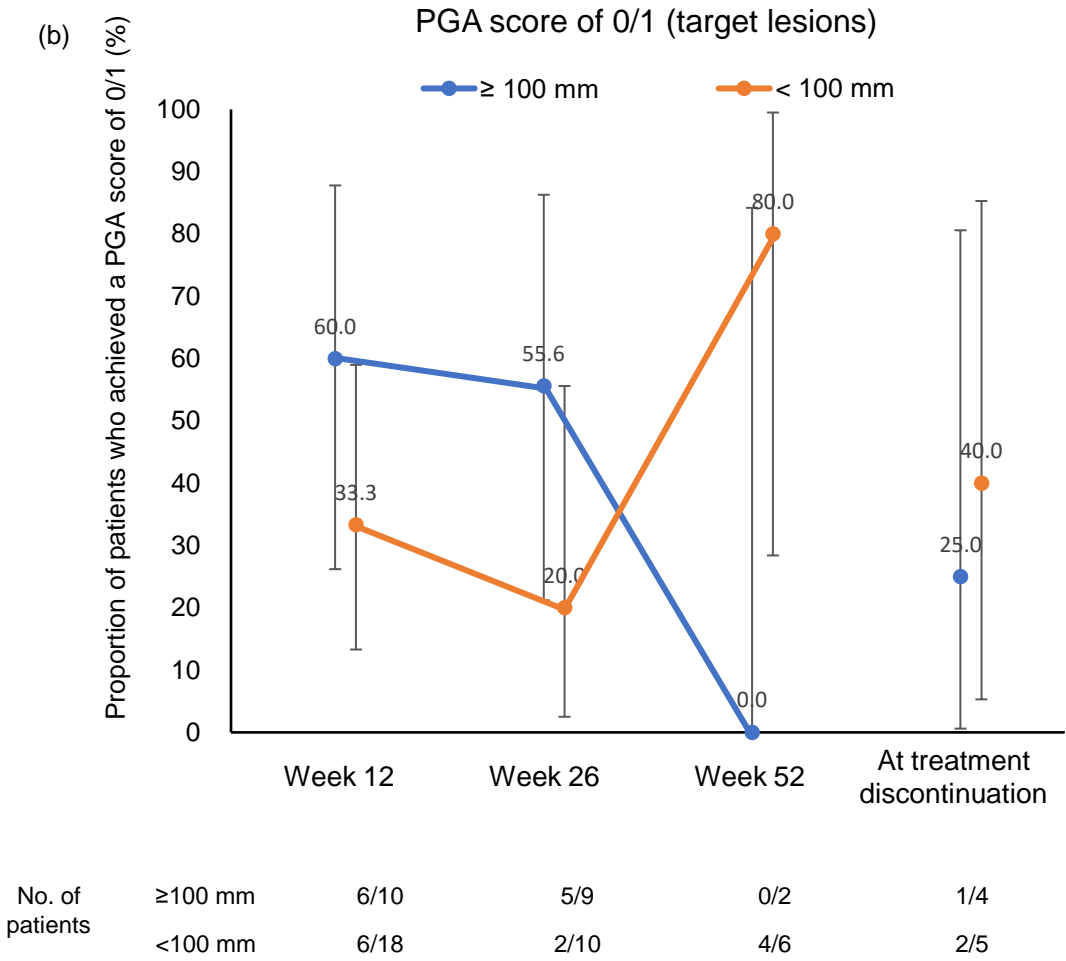

Supplement: Supplementary file 1 — Figure S1. [file JDE-52-270-s004.pdf]

Fig S3.

(a)

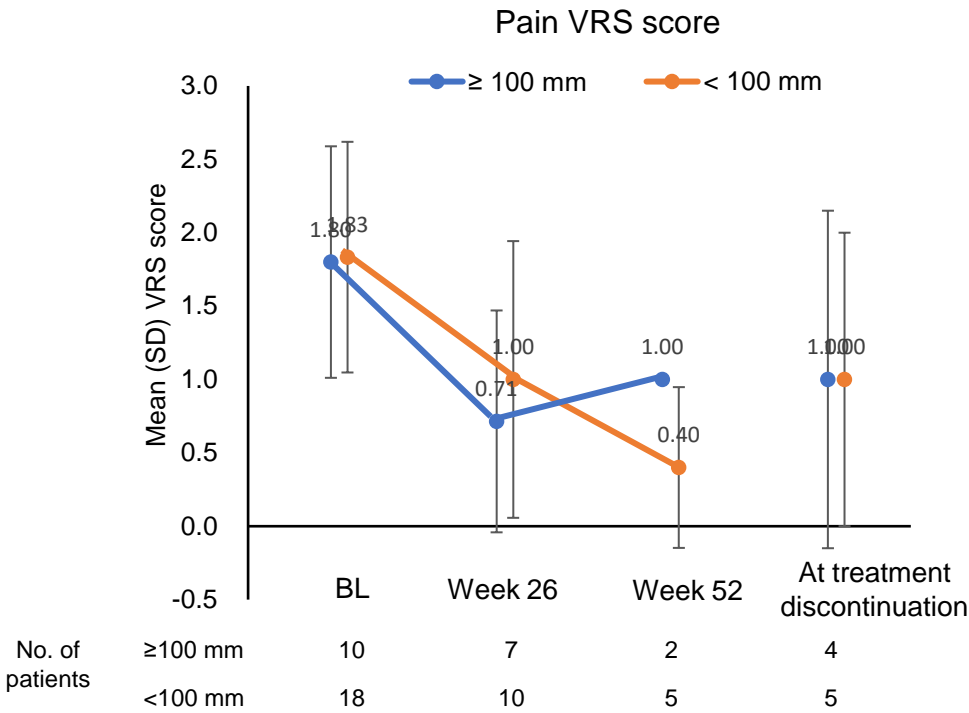

(b)

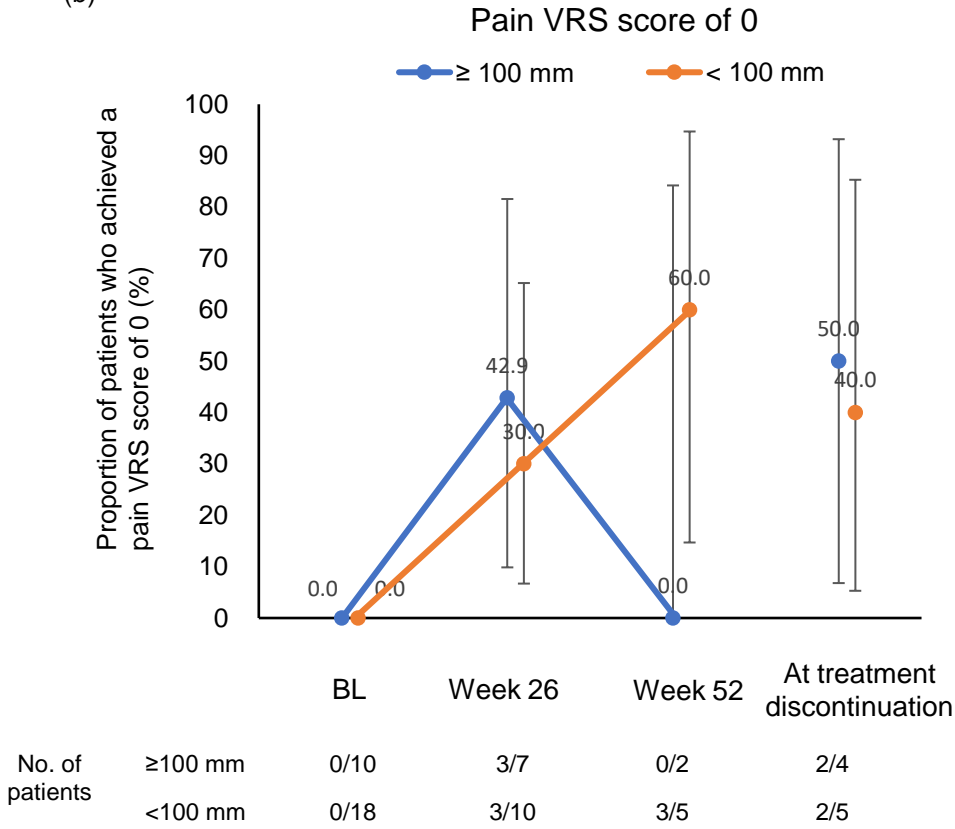

Supplement: Supplementary file 3 — Figure S3. [file JDE-52-270-s001.pdf]
