# Supplementary material for: Real‐world safety and effectiveness of adalimumab in patients with pyoderma gangrenosum: Interim analysis of a post‐marketing observational study in Japan
Source: J Dermatol. 2024 Nov 13;52(2):270–80. doi: 10.1111/1346-8138.17547 (PMC11807368; doi:10.1111/1346-8138.17547)
Supplement: Supplementary file 2 — Figure S2. [file JDE-52-270-s002.pdf]

Fig S2.

(a)

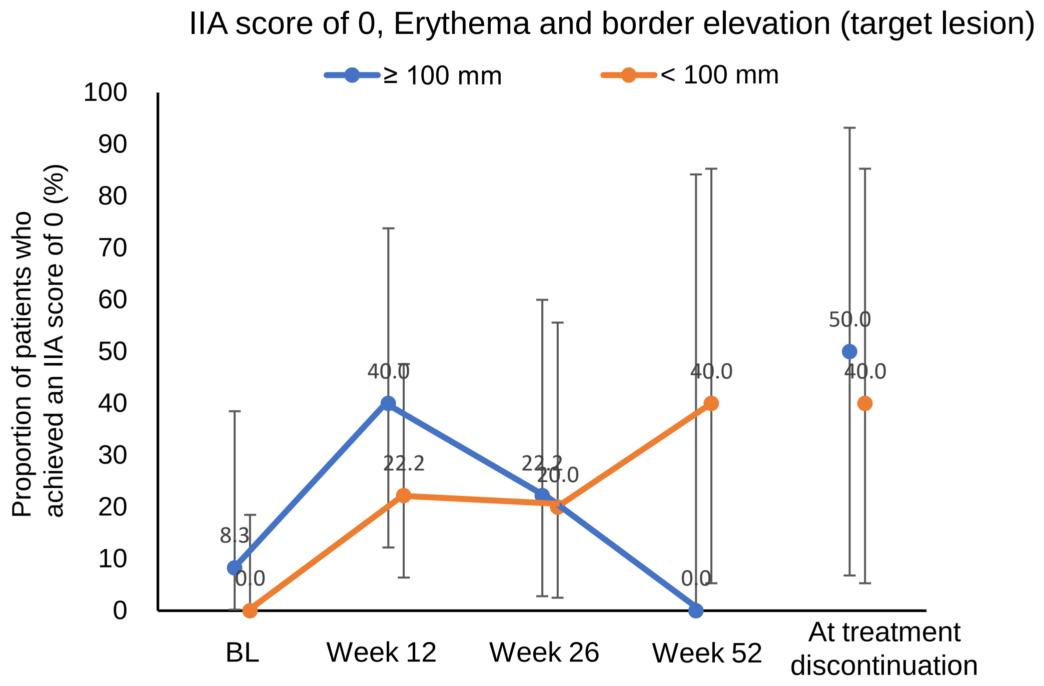

No. of patients						
	≥100 mm	1/12	4/10	2/9	0/2	2/4
	<100 mm	0/18	4/18	2/10	2/5	2/5

(b)

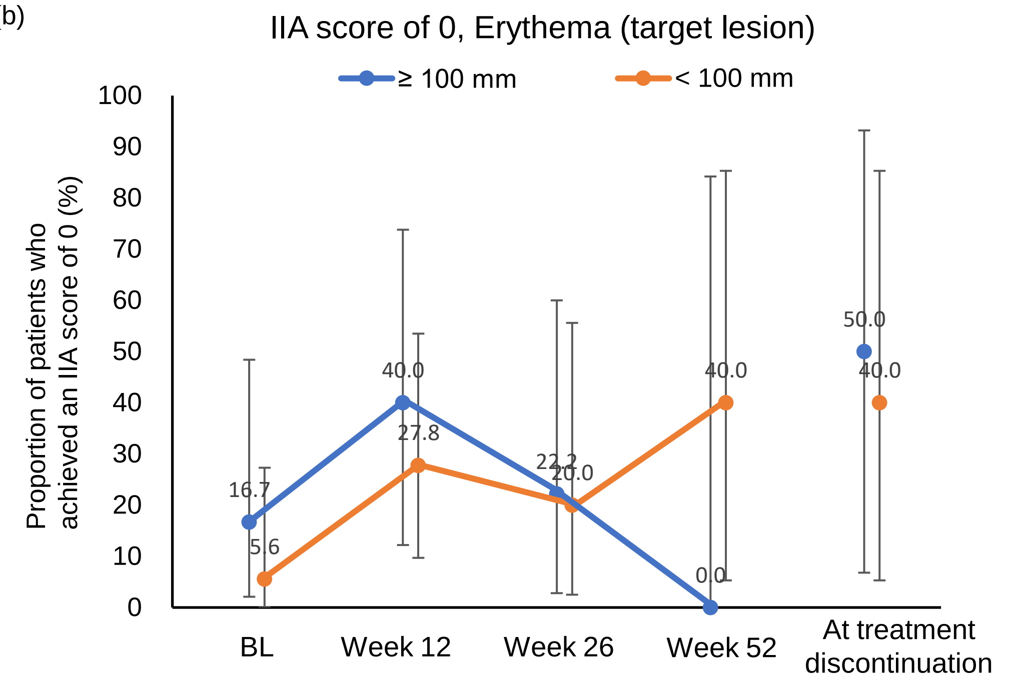

No. of patients						
	≥100 mm	2/12	4/10	2/9	0/2	2/4
	<100 mm	1/18	5/18	2/10	2/5	2/5

(c)

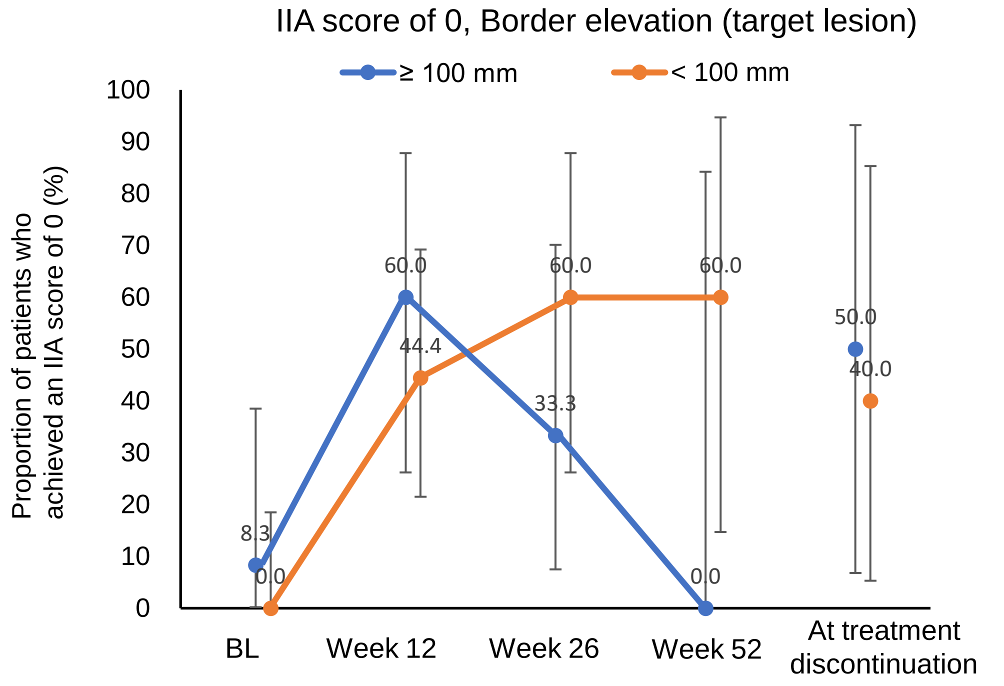

No. of patients						
	≥100 mm	1/12	6/10	3/9	0/2	2/4
	<100 mm	0/18	8/18	6/10	3/5	2/5
