# Supplementary material for: Real‐world safety and effectiveness of adalimumab in patients with pyoderma gangrenosum: Interim analysis of a post‐marketing observational study in Japan
Source: J Dermatol. 2024 Nov 13;52(2):270–80. doi: 10.1111/1346-8138.17547 (PMC11807368; doi:10.1111/1346-8138.17547)
Supplement: Supplementary file 4 — Table S1. Table S2. Table S3. Table S4. Table S5. Table S6. [file JDE-52-270-s003.docx]

**List of supporting information**

**Table S1.** Details of comorbidities

**Table S2.** Details of medical history

**Table S3.** Details of previous pharmacological treatment for PG

**Table S4.** Details of concomitant pharmacological treatment for PG

**Table S5.** Adverse events and adverse drug reactions leading to adalimumab discontinuation

**Table S6.** Proportion of patients who achieved a PGA score of 0/1, by subtype

**Table S1.** Details of comorbidities

|  | Safety analysis set  N = 37 |
| --- | --- |
| Benign, malignant, and unspecified neoplasms (including cysts and polyps) |  |
| Metastases to the liver | 1 (2.7) |
| Metastases to lymph nodes | 1 (2.7) |
| Myelofibrosis | 1 (2.7) |
| Rectal cancer | 1 (2.7) |
| Blood and lymphatic system disorders |  |
| Iron deficiency anemia | 1 (2.7) |
| Immune system disorders |  |
| Seasonal allergy | 1 (2.7) |
| Endocrine disorders |  |
| Hyperthyroidism | 1 (2.7) |
| Metabolism and nutrition disorders |  |
| Diabetes mellitus | 7 (18.9) |
| Type 2 diabetes mellitus | 3 (8.1) |
| Hyperuricemia | 2 (5.4) |
| Dyslipidemia | 2 (5.4) |
| Hyperlipidemia | 2 (5.4) |
| Steroid diabetes | 2 (5.4) |
| Hypokalemia | 1 (2.7) |
| Psychiatric disorders |  |
| Depression | 1 (2.7) |
| Nervous system disorders |  |
| Dementia | 1 (2.7) |
| Epilepsy | 1 (2.7) |
| Parkinson’s disease | 1 (2.7) |
| Eye disorders |  |
| Diabetic retinopathy | 1 (2.7) |
| Open angle glaucoma | 1 (2.7) |
| Cardiac disorders |  |
| Aortic valve incompetence | 1 (2.7) |
| Myocardial ischemia | 1 (2.7) |
| Vascular disorders |  |
| Hypertension | 4 (10.8) |
| Deep vein thrombosis | 2 (5.4) |
| Peripheral arterial occlusive disease | 2 (5.4) |
| Arteriosclerosis | 1 (2.7) |
| Arteritis | 1 (2.7) |
| Takayasu’s arteritis | 1 (2.7) |
| Varicose vein | 1 (2.7) |
| Atheroembolism | 1 (2.7) |
| Respiratory, thoracic, and mediastinal disorders |  |
| Rhinitis allergic | 1 (2.7) |
| Gastrointestinal disorders |  |
| Colitis ulcerative | 8 (21.6) |
| Crohn’s disease | 3 (8.1) |
| Gastric ulcer | 1 (2.7) |
| Retroperitoneal fibrosis | 1 (2.7) |
| Skin and subcutaneous tissue disorders |  |
| Hidradenitis | 1 (2.7) |
| Palmoplantar pustulosis | 1 (2.7) |
| Musculoskeletal and connective tissue disorders |  |
| Rheumatoid arthritis | 3 (8.1) |
| Polymyalgia rheumatica | 1 (2.7) |
| Immunoglobulin G4-related disease | 1 (2.7) |
| Destructive spondyloarthropathy | 1 (2.7) |
| Renal and urinary disorders |  |
| Chronic kidney disease | 2 (5.4) |
| Nephropathy toxic | 1 (2.7) |
| End-stage renal disease | 1 (2.7) |
| Reproductive system and breast disorders |  |
| Menopausal symptoms | 1 (2.7) |

Values are presented as n (%).

Terms were coded to primary system organ classes and preferred terms in a Japanese version of the Medical Dictionary for Regulatory Activities (version 25.1).

**Table S2.** Details of medical history

|  | Safety analysis set  N = 37 |
| --- | --- |
| Infections and infestations |  |
| Empyema | 1 (2.7) |
| Gastroenteritis | 1 (2.7) |
| Benign, malignant, and unspecified neoplasms (including cysts and polyps) |  |
| Bladder cancer | 1 (2.7) |
| Colon cancer | 1 (2.7) |
| Prostate cancer | 1 (2.7) |
| Nervous system disorders |  |
| Cerebral hemorrhage | 1 (2.7) |
| Cerebral infarction | 1 (2.7) |
| Putamen hemorrhage | 1 (2.7) |
| Vascular disorders |  |
| Embolism arterial | 1 (2.7) |
| Gastrointestinal disorders |  |
| Duodenal ulcer | 1 (2.7) |
| Large intestine perforation | 1 (2.7) |
| Skin and subcutaneous tissue disorders |  |
| Palmoplantar pustulosis | 1 (2.7) |
| Musculoskeletal and connective tissue disorders |  |
| Intervertebral disc protrusion | 1 (2.7) |
| Renal and urinary disorders |  |
| Nephrotic syndrome | 1 (2.7) |

Values are presented as n (%).

Terms were coded to primary system organ classes and preferred terms in a Japanese version of the Medical Dictionary for Regulatory Activities (version 25.1).

**Table S3.** Details of previous pharmacological treatment for PG

|  | Safety analysis set  N = 37 | |
| --- | --- | --- |
|  | Previous treatment before adalimumab initiation | Previous treatment immediately before adalimumab initiation |
| **Systemic therapy** |  |  |
| Steroids | 26 (70.3) | 26 (70.3) |
| Analgesics (e.g., NSAIDs) | 13 (35.1) | 13 (35.1) |
| Antibiotics/antibacterial drugs | 7 (18.9) | 6 (16.2) |
| Cyclosporine | 5 (13.5) | 1 (2.7) |
| Methotrexate | 2 (5.4) | 2 (5.4) |
| Salazosulfapyridine | 1 (2.7) | 1 (2.7) |
| Mesalazine | 1 (2.7) | 1 (2.7) |
| Biologics | 1 (2.7) | 0 (0.0) |
| Other | 9 (24.3) | 6 (16.2) |
| **Local therapy** |  |  |
| Steroids | 18 (48.6) | 14 (37.8) |
| Debridement agents | 9 (24.3) | 8 (21.6) |
| Tissue regeneration accelerators | 6 (16.2) | 6 (16.2) |
| Antibiotics/antibacterial drugs | 6 (16.2) | 5 (13.5) |
| Tacrolimus | 2 (5.4) | 1 (2.7) |
| Other | 10 (27.0) | 8 (21.6) |

Values are presented as n (%).

NSAIDs, nonsteroidal anti-inflammatory drugs; PG, pyoderma gangrenosum.

**Table S4.** Details of concomitant pharmacological treatment for PG

|  | Safety analysis set  N = 37 | | |
| --- | --- | --- | --- |
| Treatment | **Concomitant treatment initiated at any time** | **Concomitant treatment initiated before adalimumab treatment** | **Concomitant treatment initiated during adalimumab treatment** |
| **Systemic therapy** |  |  |  |
| Steroids | 21 (56.8) | 20 (54.1) | 5 (13.5) |
| Analgesics (NSAIDs, etc.) | 10 (27.0) | 10 (27.0) | 0 (0.0) |
| Antibiotics/antibacterial drugs | 2 (5.4) | 1 (2.7) | 1 (2.7) |
| Cyclosporine | 1 (2.7) | 0 (0.0) | 1 (2.7) |
| Mesalazine | 1 (2.7) | 1 (2.7) | 0 (0.0) |
| Diphenyl sulfone | 1 (2.7) | 1 (2.7) | 0 (0.0) |
| Potassium iodide | 1 (2.7) | 1 (2.7) | 0 (0.0) |
| Salazosulfapyridine | 1 (2.7) | 1 (2.7) | 0 (0.0) |
| Alprostadil | 1 (2.7) | 0 (0.0) | 1 (2.7) |
| Polyethylene glycol-treated human Intravenous immunoglobulin | 1 (2.7) | 0 (0.0) | 1 (2.7) |
| **Local therapy** |  |  |  |
| Steroids | 9 (24.3) | 6 (16.2) | 3 (8.1) |
| Antibiotics/antibacterial drugs | 6 (16.2) | 5 (13.5) | 2 (5.4) |
| Trafermin (recombinant) | 2 (5.4) | 1 (2.7) | 1 (2.7) |
| Heparinoid | 1 (2.7) | 1 (2.7) | 1 (2.7) |
| Bucladesine sodium | 1 (2.7) | 0 (0.0) | 1 (2.7) |
| Iodine | 1 (2.7) | 1 (2.7) | 0 (0.0) |
| Alprostadil alfadex | 1 (2.7) | 0 (0.0) | 1 (2.7) |
| Tacrolimus hydrate | 1 (2.7) | 1 (2.7) | 0 (0.0) |

Values are presented as n (%).

Systemic analgesics include acetaminophen, loxoprofen sodium hydrate, tramadol hydrochloride, and tramadol hydrochloride/acetaminophen.

Systemic steroids include betamethasone and prednisolone.

Systemic antibiotics/antibacterial drugs include doxycycline hydrochloride and trimethoprim-sulfamethoxazole.

Local antibiotics include silver sulfadiazine, gentamicin sulfate, polymyxin B sulfate, and a mixture of sugar and povidone-iodine.

Local steroids include clobetasol propionate, betamethasone butyrate propionate, and hydrocortisone.

NSAIDs, nonsteroidal anti-inflammatory drugs; PG, pyoderma gangrenosum.

**Table S5.** Adverse events and adverse drug reactions leading to adalimumab discontinuation (safety analysis set, N = 37)

|  | AEs | Serious AEs | ADRs | Serious ADRs |
| --- | --- | --- | --- | --- |
| Incidence proportion | **7 (18.9)** | **6 (16.2)** | **5 (13.5)** | **4 (10.8)** |
| Number of events | 10 | 8 | 8 | 6 |
| Infections and infestations | **5 (13.5)** | **5 (13.5)** | **4 (10.8)** | **4 (10.8)** |
| Pneumonia | 2 (5.4) | 1 (2.7) | 2 (5.4) | 1 (2.7) |
| Pneumonia aspiration | 1 (2.7) | 1 (2.7) | 0 (0.0) | 0 (0.0) |
| Sepsis | 1 (2.7) | 1 (2.7) | 1 (2.7) | 1 (2.7) |
| Tuberculosis | 1 (2.7) | 1 (2.7) | 1 (2.7) | 1 (2.7) |
| Urinary tract infection | 1 (2.7) | 1 (2.7) | 1 (2.7) | 1 (2.7) |
| Cytomegalovirus enterocolitis | 1 (2.7) | 1 (2.7) | 1 (2.7) | 1 (2.7) |
| Nervous system disorders |  |  |  |  |
| Cerebral infarction | 1 (2.7) | 1 (2.7) | 1 (2.7) | 1 (2.7) |
| Cardiac disorders |  |  |  |  |
| Cardiac failure | 1 (2.7) | 1 (2.7) | 0 (0.0) | 0 (0.0) |
| Hepatobiliary disorders |  |  |  |  |
| Abnormal hepatic function | 1 (2.7) | 0 (0.0) | 1 (2.7) | 0 (0.0) |

Values are presented as n (%) or n.

AEs and ADRs were coded to primary system organ classes and preferred terms in a Japanese version of the Medical Dictionary for Regulatory Activities (version 25.1).

ADRs, adverse drug reactions; AEs, adverse events.

**Table S6.** Proportion of patients who achieved a PGA score of 0/1 for total lesions, by subtype

| Subtype | Week 12 | Week 26 | Week 52 | At treatment discontinuation |
| --- | --- | --- | --- | --- |
| Ulcerative, excluding peristomal | 9/23 | 6/15 | 3/6 | 2/7 |
| Peristomal | 0/1 | 0/1 | – | – |
| Pustular | 2/2 | 1/1 | – | 1/2 |
| Vegetative | 1/2 | 0/2 | 1/2 | – |

PGA, physician global assessment.
